# Solid-Contact Potentiometric Anion Sensing Based on Classic Silver/Silver Insoluble Salts Electrodes without Ion-Selective Membrane

**DOI:** 10.3390/membranes11120959

**Published:** 2021-12-05

**Authors:** Chunxian Liao, Lijie Zhong, Yitian Tang, Zhonghui Sun, Kanglong Lin, Longbin Xu, Yan Lyu, Dequan He, Ying He, Yingming Ma, Yu Bao, Shiyu Gan, Li Niu

**Affiliations:** 1 Guangzhou Key Laboratory of Sensing Materials & Devices, Center for Advanced Analytical Science, School of Chemistry and Chemical Engineering, Guangzhou University, Guangzhou 510006, China; gdcxliao@e.gzhu.edu.cn (C.L.); ccljzhong@gzhu.edu.cn (L.Z.); yitiantang@e.gzhu.edu.cn (Y.T.); cczhsun@gzhu.edu.cn (Z.S.); longkanglin@e.gzhu.edu.cn (K.L.); longbinx@gzhu.edu.cn (L.X.); yanlyu@e.gzhu.edu.cn (Y.L.); dequanhe@e.gzhu.edu.cn (D.H.); ccyhe@gzhu.edu.cn (Y.H.); ccymma@gzhu.edu.cn (Y.M.); baoyu@gzhu.edu.cn (Y.B.); lniu@gzhu.edu.cn (L.N.); 2 School of Civil Engineering, Guangzhou University, Guangzhou 510006, China

**Keywords:** potentiometric sensing, anion sensors, ion-selective electrodes, wearable ion sensors

## Abstract

Current solid potentiometric ion sensors mostly rely on polymeric-membrane-based, solid-contact, ion-selective electrodes (SC-ISEs). However, anion sensing has been a challenge with respect to cations due to the rareness of anion ionophores. Classic metal/metal insoluble salt electrodes (such as Ag/AgCl) without an ion-selective membrane (ISM) offer an alternative. In this work, we first compared the two types of SC-ISEs of Cl^−^ with/without the ISM. It is found that the ISM-free Ag/AgCl electrode discloses a comparable selectivity regarding organic chloride ionophores. Additionally, the electrode exhibits better comprehensive performances (stability, reproducibility, and anti-interference ability) than the ISM-based SC-ISE. In addition to Cl^−^, other Ag/AgX electrodes also work toward single and multi-valent anions sensing. Finally, a flexible Cl^−^ sensor was fabricated for on-body monitoring the concentration of sweat Cl^−^ to illustrate a proof-of-concept application in wearable anion sensors. This work re-emphasizes the ISM-free SC-ISEs for solid anion sensing.

## 1. Introduction

On-site ion analysis and monitoring in complex environments require miniaturized and integrated devices. The representative electrochemical ion analytical methods are focused on ion-selective electrodes (ISEs) [1] and ion transfer at the interface between immiscible electrolyte solutions (ITIES) [2]. The classic ISEs are the liquid-junction system, which is limited to miniaturization due to an inner filling solution. ITIES electrochemistry can be used for an ion-sensing platform by tuning an externally applied potential. Either option faces the possibility of miniaturization and integration. Solid-contact ion-selective electrodes (SC-ISEs) are developed from the classic liquid-junction ISEs, which satisfy the requirement of on-site ion analysis [3,4,5,6,7]. Since the concept of SC-ISEs [8] was first proposed in 1971, this solid-state potentiometric sensing has been expanded from detection of ions to molecules [9,10], biomolecules [11,12,13,14], and even bacteria [15,16]. The basic structure is composed of a sandwich model, including two layers of solid contact (ion-to-electron signal transduction) and ion-selective membrane (ISM, ion recognition). There are two long-lasting challenges to this structure. One is the potential stability and/or reproducibility caused by the ultrathin water layer and interfacial capacitance between solid contact and ISM [17,18,19,20]. Numerous efforts have been devoted to improving the properties of SC materials (such as hydrophobicity and redox capacitance with defined phase boundary potential) to address these issues [19,20,21,22,23,24,25]. The other is the leaking of ISM components leading to biological toxicity [26], particularly for medical applications. In addition, the weak mechanical strength of the organic ISM results in a short life in a complex environment.

Currently, most of SC-ISEs have focused on the detection of cations, while there are only a few reports for anion sensing. The early reported anion SC-ISEs are based on plastic ion exchangers without ionophores [27,28,29,30,31,32,33,34]. For example, poly (vinyl chloride)-matrix (PVC) membrane containing chlorate ion exchanger was proposed for solid-state chlorate ISE [27]. Since the ISM was prepared through ion exchange (such as a nitrate ion exchanger), the selectivity ability toward interfering nitrate is rather low. Later, ionophore-based anion SC-ISEs have been developed [35,36,37,38,39,40]. For example, 4-(4-bromophenyl)-2,6-diphenylpyrilium perchlorate (BDPP) was synthesized for solid-state sulfate sensing [35], which exhibits improved selectivity and a low detection limit. Recently, multiwalled carbon nanotube as the SC layer coupled with anion-selective ionophores has realized sensing toward a few anions in seawater [39]. To regulate the selectivity and sensitivity, porphyrin dimers through molecular bridge connection have been recently proposed as promising anion/cation acceptors (such as ClO_4_^−^) compared to a traditional single porphyrin unit [40]. Another classic potentiometric anion sensor is based on metal/metal insoluble salts electrodes without ISM, typically like the Ag/AgCl electrode for Cl^−^ sensing [41,42,43,44,45,46]. It relies on an equilibrium between AgCl and Cl^−^ (AgCl (s) + e^−^ ↔ Cl^−^ (aq) +Ag (s)). The unique lattice of silver chloride makes Cl^−^ exchange between solid AgCl and the solution, which is the origin of a specific response to Cl^−^. The Ag/AgCl electrode has been used for environmental Cl^−^ detection, for example, by using a screen-printed technique [43]. Other metal/metal insoluble salts electrodes, typically like Ag/Ag_2_S [47] and Pb/PbSiO_3_ [48] without ISM, have been demonstrated for sensing toward S^2−^ and silicate, respectively. Early studies indicate the possibility of constructing the ISM-free SC-ISEs. Recently, we presented a new type of SC-ISEs without using the ISM based on classic Li-ion battery materials [49]. The materials (such as LiFePO_4_ and LiMn_2_O_4_) as solid contact layers can realize both functions of ion-to-electron transduction and ion recognition.

In this work, we first compare two types of SC-ISEs of Cl^−^ with ISM and without ISM. It is found that the ISM-free Ag/AgCl electrode discloses a comparable selectivity regarding ISM-based SC-ISE of Cl^−^, and much better performances of response time, stability, reproducibility, and anti-interferences from gas and light. More importantly, these silver/silver insoluble salts electrodes are also feasible for other single and multi-valent anions sensing. Moreover, a wearable Cl^−^ sensor based on the Ag/AgCl electrode was fabricated. The electrode maintained mechanical flexibility and analytical performances under the bending state. The sweat Cl^−^ concentration was monitored by a flexible wearable sensor. Through this work, we re-emphasize the ISM-free SC-ISEs for solid anion sensing.

## 2. Materials and Methods

### 2.1. Reagents

Potassium tetrakis(pentafluorophenyl)borate (KTPFB, 97%) was purchased from Alfa (Shanghai, China). High-molecular-weight poly(vinyl chloride) (PVC) (Selectophore), tridodecylmethylammonium chloride (TDMACl, 98%), Bis(2-ethylhexyl) sebacate (DOS, ≥97%), Chloride ionophore I (Selectophore), and tetrahydrofuran (THF) (anhydrous, ≥99%, inhibitor-free) were obtained from Sigma-Aldrich (Shanghai, China). All the reagents were used directly after receiving, and then using ultrapure water (18.2 MΩ·cm) to prepare aqueous solutions.

### 2.2. Preparation of Electrodes

The silver electrodes with a diameter of 3 mm were polished with 1 μm and 0.3 μm alumina powder, then were ultrasonically and successively cleaned with water-ethanol-water. The silver electrode was then placed in 3 M KCl aqueous solution and deposited with a thin layer of gray–black silver chloride in a constant current of 25 μA for 90 min. This was the preparation procedure for the Ag/AgCl electrode. The ISM electrode was obtained by dropping 20 μL Cl^−^ selective membrane solution onto the Ag/AgCl electrode. The Cl^−^ selective membrane solution was obtained by dissolving 250 mg of the membrane cocktail in 2.5 mL of THF: 32.9 wt % PVC and 65.7 wt % DOS, 1 wt % Chloride ionophore I, and 0.4 wt % TDMACl. The membrane solution was stored at 4 °C. Before the test, the Ag/AgCl electrode without ISM was conditioned in a 10^−7^ M KCl aqueous solution for 2 h. The ISM electrode was firstly balanced in 10^−4^ M KCl aqueous solution overnight, and then conditioned in a 10^−7^ M KCl aqueous solution for 3 h before the test.

### 2.3. Preparation of Flexible Electrodes

A poly(ethylene terephthalate) (PET) substrate of 7 × 7 cm^2^ was cleaned successively with water-acetone-isopropanol, and then was etched with O_2_ plasma for 2 min. Then, the PET substrate was sputtered with 30-40-200 nm chrome-gold-silver electrode (5 mm diameter) by using ultra-high vacuum sputtering technology (AJA Orion 5, Scituate, MA, USA). After coating with a polydimethylsiloxane (PDMS) insulating layer, the flexible electrode was dried at 90 °C for 50 min. The flexible Ag/AgCl electrode was obtained by oxidizing the silver electrode in 0.05 M FeCl_3_ for 10 s. For the preparation of the all-solid-state reference electrode (RE), the PET-supported chrome-gold-silver electrode was first oxidized in 0.05 M FeCl_3_ for 10 s to form Ag/AgCl. Then, 20 μL reference membrane (RM) solution was drop-casted on the Ag/AgCl and dried overnight in ambient conditions. The RM solution was prepared by dissolving 500 mg of the membrane cocktail in 5 mL of THF: 150 mg PVC and 340 mg DOS, 5.5 mg TDMACl, 4.5 mg KTPFB, 808 mg KCl, and 306 mg AgCl powder were mixed and stirred overnight at ambient temperature [50,51]. After the RM dried, a reference protection layer was further covered on the RM. The protection layer was prepared by dissolving 80 mg of the cocktail in 1 mL of THF: 26.48 mg PVC and 53.52 mg DOS. After coating, the electrode was dried at ambient temperature overnight. The principle for the all-solid-state RE is the same as the liquid-junction RE but with a solid electrolyte membrane instead. Before the test, the flexible Ag/AgCl electrode was conditioned in 10^−7^ M KCl aqueous solution for 2 h, while the RE was balanced in 3 M KCl aqueous solution overnight.

### 2.4. Electrochemical Characterization of SC-ISEs

All electrochemical performances of the electrodes were characterized by a Gamry electrochemical workstation (reference 600 plus, Gamry, Warminster, PA, USA) and multi-channel potentiometer EMF6 (Lawson Lab, Inc., Malvern, PA, USA) at ambient conditions. The on-body sweat analysis was carried out by a home-made mini-potentiometer with an input resistance of 10^13^ Ω. The electrode concentration-response curves (including determination of selectivity coefficient) were measured in the aqueous solutions (10^−7^ M to 10^−1^ M), and the Debye-Hückel equation was used to correct the ion activities. Other performance characterizations were performed, including chronopotentiometry (±1 nA current applied), water layer, and anti-interference experiments. A commercial Hg/Hg_2_Cl_2_/saturated KCl/1 M LiOAc (Tjaida, Tianjing, China) was used as a reference electrode, and a platinum wire was used as the auxiliary electrode.

### 2.5. Sweat Analysis

A commercial sweat guide band was used to fix the prepared flexible electrode and to enrich the sweat. Combining with a multi-channel mini-potentiometer (home-made) and wireless data transmission mode, the chloride ion concentration in the sweat produced by the volunteer during running was analyzed. Before starting the run, the volunteer’s forehead was wiped with medical alcohol and deionized water in turn. Then, the volunteer ran at a graded load on the treadmill for a period and used a microtubule to collect the sweat sample after cooling down. After the concentration of Cl^−^ in sweat was in situ analyzed by using the flexible wearable Ag/AgCl electrode, the sweat sample was diluted and analyzed with an ion chromatograph. It should be noted that the volunteer agrees to this test of wearable sensors for sweat analysis.

## 3. Results and Discussion

### 3.1. Potentiometric Responses of SC-ISEs of Cl^−^ with/without ISM

**Figure 1:**
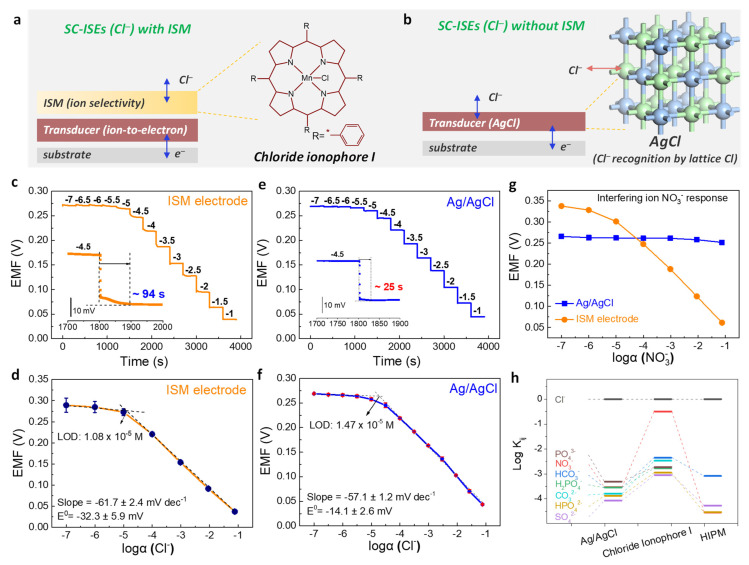
Potentiometric responses of ion−selective membrane (ISM) electrode and Ag/AgCl electrode toward Cl^−^. (**a**,**b**) A schematic diagram illustrates two types of solid-contact ion-selective electrodes (SC−ISEs) of Cl^−^ including (**a**) ISM−based sandwich structure supported by Ag/AgCl solid contact and (**b**) ISM−free structure of bare Ag/AgCl. Chloride ionophore I was used for Cl^−^ recognition in ISM. (**c**,**d**) Potentiometric response and calibration curve of the ISM electrode from 0.1 μM to 0.1 M Cl^−^. The inset shows the response time. It should be noted that the response time is defined at the electromotive force (EMF) with a difference of 0.5 mV from the final steady EMF. (**e**,**f**) Potentiometric response and calibration curve of the Ag/AgCl from 0.1 μM to 0.1 M Cl^−^. The inset shows the response time. (**g**) Potentiometric responses of ISM electrode and Ag/AgCl toward an example of interfering ion NO_3_^−^. (**h**) Comparison of selectivity coefficients towards a series of interfering ions. The coefficients were measured by the separation solution method. All the error bars shown in the figures indicate three individual electrodes. The data for the Cl^−^ receptor of 2− (1-H-imidazo [4,5-f] [1,10]-phenanthroline-2-yl) −6methoxyphenol (HIPM) is adapted from reference [52].

Two types of typical SC-ISEs (Cl^−^) are shown in Figure 1a,b. One is the three-layer structure based on an ISM containing chloride ionophore I (Figure 1a). It should be noted that the solid contacts could be other materials, such as the classic poly (3,4-ethylenedioxythiophene) (PEDOT) or carbon-based materials. Herein, the Ag/AgCl was chosen as the SC layer for a direct comparison. The other is bare Ag/AgCl without ISM (Figure 1b). We first compare the potentiometric responses of the ISM electrode and Ag/AgCl electrode toward Cl^−^. The potential response curves of both electrodes were measured in KCl solution (range from 10^−7^ to 10^−1^ M) with three separately prepared electrodes. As shown in Figure 1c, the ISM electrode shows a response starting from ~10^−5^ M with a response time around 94 s (the inset). This slow potential equilibrium limits the application in rapid on-site detection. Its calibration curve exhibits the sensitivity of −61.7 ± 2.4 mV dec^−1^ and standard E^0^ of −32.3 ± 5.9 mV within the linear response range ~10^−5^ to 10^−1^ M (Figure 1d). The limit of detection (LOD) of ISM electrode for Cl^−^ is 1.08 × 10^−5^ M. For comparison, the ISM-free Ag/AgCl electrode exhibits a similar potentiometric response toward Cl^−^ (Figure 1e) with a Nernst slope of −57.1 ± 1.2 mV dec^−1^ and a close LOD of 1.47 × 10^−5^ M (Figure 1f). The response time for Ag/AgCl of ~25 s (the inset, Figure 1e) is much faster than the ISM electrode. It should be noted that the response time is defined at the electromotive force (EMF) with a difference of 0.5 mV from the final steady EMF. In addition, the E^0^ of −14.1 ± 2.6 mV showed a lesser standard deviation (Figure 1f), which indicates a better reproducibility.

In addition to the target ion response, another crucial parameter for SC-ISEs is the selectivity toward interfering ions. The separation solution method was used to determine the selectivity toward other anions for both electrodes. Taking interfering anion NO_3_^−^ as an example, the potential response curves of Ag/AgCl and ISM electrodes to nitrate ion were measured in KNO_3_ solution with concentrations ranging from 10^−7^ to 10^−1^ M (Figure 1g). It was found that the Ag/AgCl electrode had almost no response during the whole concentration range (blue line, Figure 1g), while the ISM electrode disclosed a Nernst response toward NO_3_^−^ (orange line, Figure 1g). Through calculation, the selectivity coefficients (log *K*_ij_) toward NO_3_^−^ were determined to be −3.53 ± 0.03 for Ag/AgCl and −0.49 ± 0.19 for the ISM electrode. The selectivity improved for nearly three orders of magnitudes. Other interfering ion measurements are shown in Figure S1. All selectivity coefficients are summarized in Figure 1h. It was surprisingly found that the selectivity of the Ag/AgCl electrode to Cl^−^ is much better than that of the ISM electrode. Except for PO_4_^3−^, the selectivity toward other interfering ions is at least one magnitude higher than the organic chloride ionophore I.

It should be noted that chloride ionophore I was chosen as the ionic carrier due to it being a commercial chemical and having a relatively low cost. We also compared the selectivity of Ag/AgCl with a representative lab-synthesized anion receptor, 2-(1-H-imidazo [4,5-f] [1,10]-phenanthroline-2-yl)-6methoxyphenol (HIPM) [52]. The HIPM shows the log *K*_ij_ = −4.54 (NO_3_^−^), −3.08 (HCO_3_^−^), −4.27 (SO_4_^2−^), and −4.53 (HPO_4_^2−^) (Figure 1h). Regarding HIPM, Ag/AgCl exhibits relatively less selectivity toward NO_3_^−^ but comparable selective abilities toward other anions. We also compared the analytical performances of Ag/AgCl electrodes with previously reported SC-ISEs of Cl^−^ sensors (Tables S1 and S2, Supporting Information). It is found that the as-prepared ISM-free Ag/AgCl discloses comparable or even better comprehensive performances compared with/without ISM-based Cl^−^ sensors. Overall, the above results demonstrated the Nernst response, rapid response time, good selectivity, and reproducibility for the ISM-free Ag/AgCl. Other important electrochemical performances are further evaluated, including stability and anti-light and gas abilities.

### 3.2. Comparison of Stability and Anti-Interference

**Figure 2:**
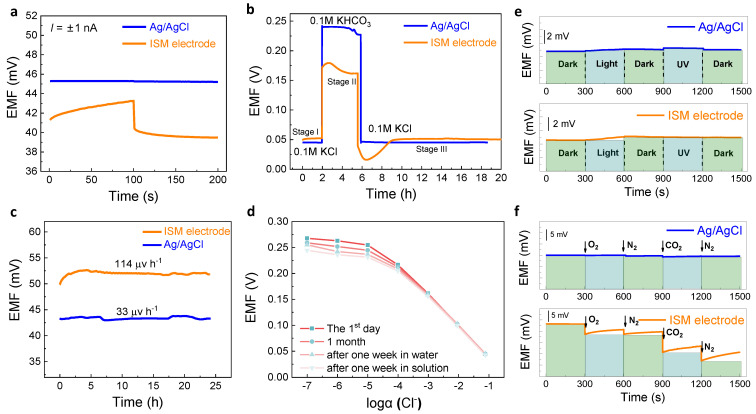
Stability and anti−interference of Ag/AgCl electrode and ISM electrode. (**a**) Chronopotentiometry measurements in 0.1 M KCl at the current (*I*) of ±1 nA. (**b**) Water−layer tests for both electrodes. (**c**) Medium−term stability of the Ag/AgCl electrode and the ISM electrode in 0.1 M KCl. (**d**) Long−term stability of the Ag/AgCl electrode under different storage conditions. (**e**,**f**) Light− and gas−sensitive examinations.

Another crucial basis for the SC-ISEs is the potential stability. A short-term potential stability of the electrodes was first evaluated using reversed-current chronopotentiometry. According to the typical chronopotentiogram in Figure 2a, when the currents of +1 and −1 nA were applied to the two electrodes for 100 s, the potential change of the ISM electrode (14.67 μV s^−1^) was significantly greater than that of the Ag/AgCl electrode (0.31 μV s^−1^). The interfacial capacitances can be calculated according to the equation ΔE/Δt = *i*/C. The Ag/AgCl and ISM electrodes show the capacitances of 3.22 mF and 0.068 mF, respectively. Obviously, the Ag/AgCl exhibits ~50-folders higher capacitance so that a lower potential drift resulted compared with the ISM electrode. Another possible reason for the potential drift of the ISM electrode was the introduction of a layer of ISM, leading to the formation of a water layer at the SC/ISM interface. To examine this effect, the water layer tests are further performed.

The potentiometric test for the water-layer examination was firstly measured in 0.1 M KCl, then in 0.1 M KHCO_3_, and again back in 0.1 M KCl (Figure 2b). The ISM electrode reveals a significant potential drift, particularly at stage II, i.e., upon transferring from KCl to interfering ion solution (KHCO_3_) (orange line, Figure 2b). At stage III, the potential undergoes ~3 h equilibrium back to the stable potential. For comparison, the potential response of the Ag/AgCl electrode reaches equilibrium quickly (blue line, Figure 2b). Furthermore, the medium-term stability (24 h) was examined (Figure 2c). The potential drift of the Ag/AgCl electrode was only 33 μV h^−1^, while the ISM electrode was 114 μV h^−1^. In addition, the long-term stability of the Ag/AgCl electrode was tested by placing the electrode in the ambient conditions for one month and soaking it in water and KCl solution for one week, respectively. As shown in Figure 2d, the calibration curves of the treated electrodes are nearly overlapped within the range of linear response concentration.

The gas and light interferences were finally examined. According to Figure 2e, both electrodes are almost unaffected by light. However, the gases (such as O_2_ and CO_2_) cause potential fluctuations for the ISM electrode due to a water-layer formation at the SC/ISM interface (Figure 2f). In the water layer, the O_2_ forms a half-battery, and CO_2_ changes the pH, leading to significant potential drift, which was also demonstrated by the water-layer tests (Figure 2b). In contrast, the ISM-free Ag/AgCl shows nearly no interference from gas. Overall, we demonstrate that the stability of the Ag/AgCl electrode is significantly improved compared with the ISM electrode.

### 3.3. Ag/AgX-Based SC-ISEs for Various Anion Sensing

**Figure 3:**
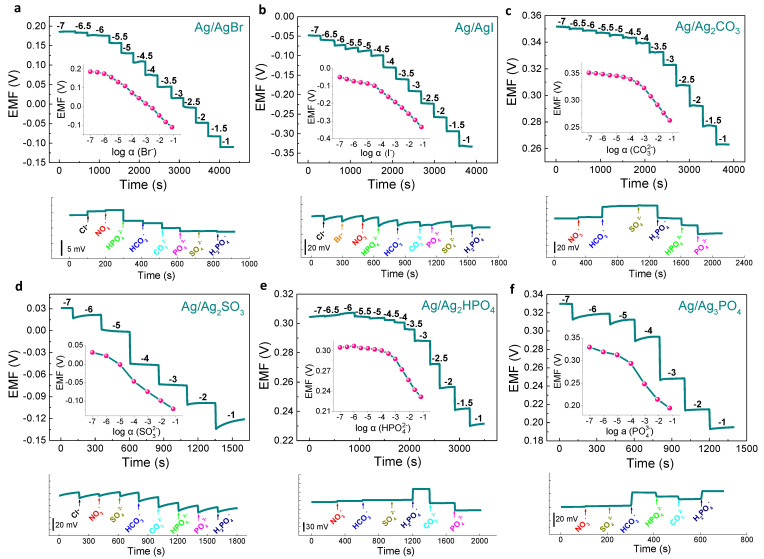
ISM−free Ag/AgX−based SC−ISEs for anion sensing. Potentiometric response curves toward various valence−state anions from 0.1 μM to 0.1 M: (**a**) Ag/AgBr electrode for Br^−^, (**b**) Ag/AgI electrode for I^−^, (**c**) Ag/Ag_2_CO_3_ electrode for CO_3_^2−^, (**d**) Ag/Ag_2_SO_3_ for SO_3_^2−^, (**e**) Ag/Ag_2_HPO_4_ for HPO_4_^2−^, and (**f**) Ag/Ag_3_PO_4_ electrode for PO_4_^3−^. All the insets shown in the figures represent corresponding calibration curves. Interference tests are shown under the response curves.

The above results demonstrated that the ISM-free Ag/AgCl has a Nernst response, high selectivity, good stability, and reproducibility. This result inspires us to develop other Ag/AgX-based, anion-selective electrodes. Like the method for preparing Ag/AgCl electrodes, the polished silver electrode was placed in the corresponding anionic potassium salt solution, and a constant current was applied for 90 min electrodeposition to obtain the Ag/AgX electrodes. Subsequently, the potential responses and their calibration curves were obtained. Moreover, the selectivity was examined by continually adding 0.1 M interference ion solution to 0.1 M target ion solution. All measurement results are shown in Figure 3 (insets show the calibration curves). The potential responses of Ag/AgBr (Figure 3a) and Ag/AgI (Figure 3b) electrodes with the same valence as Cl^−^ are close to the Nernst response, with slopes of −59.6 mV dec^−1^ and −64.7 mV dec^−1^, and the LODs are 10^−5.82^ M and 10^−4.61^ M, respectively.

It is worth noting that their potentials were basically stable and unaffected when other anion interferences were added. Similarly, the potential responses of other divalent anions, such as the Ag/Ag_2_CO_3_ electrode (Figure 3c), Ag/Ag_2_SO_3_ electrode (Figure 3d), and Ag/Ag_2_HPO_4_ electrode (Figure 3e) to their respective anions, were basically consistent with Nernst responses, and their detection limits can also reach a relatively low concentration. For the trivalent anion, the Ag/Ag_3_PO_4_ electrode exhibits a slope of −35.6 mV dec^−1^, which is higher than the theoretical value of the Nernst response (Figure 3f). The possible reason is that the trivalent PO_4_^3−^ can be hydrolyzed, resulting in the presence of both monovalent and divalent phosphate in the solution. Therefore, the response of Ag/Ag_3_PO_4_ electrode to trivalent PO_4_^3−^ did not fully correspond to the Nernst slope. In the aspect of anti-interference experiment results, the divalent and trivalent Ag/AgX electrodes were relatively affected by some anions, which may be mainly due to the hydrolysis between target ions and the co-ion effects. Overall, the above results confirm the feasibility of using insoluble silver salts as solid contact layers to prepare ISM-free anion SC-ISEs.

### 3.4. Wearable Sensor for Sweat Analysis

**Figure 4:**
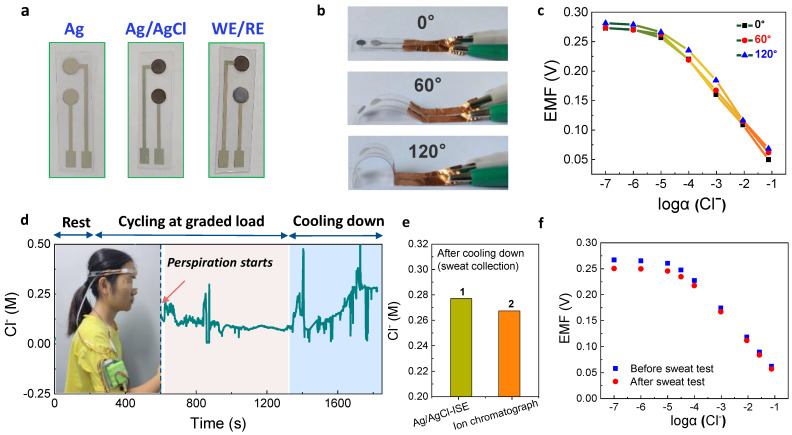
Flexible Ag/AgCl electrode toward on−body analysis of Cl^−^. (**a**) Optical images of the preparation of flexible Ag/AgCl electrode. The working electrode (WE) is Ag/AgCl, and the reference electrode (RE) is solid Ag/AgCl/PVC−KCl. (**b**) Optical images of flexible Ag/AgCl electrode under different bending angles. (**c**) Calibration curves of flexible Ag/AgCl electrode at different bending angles. (**d**) In situ monitoring curve of sweat Cl^−^. A volunteer wearing the prepared wearable Cl^−^ sensor during running. (**e**) A comparison of ex situ sweat Cl^−^ detection by ion chromatograph. The sweat was collected after cooling down and diluted for the measurements. (**f**) Calibration curves of flexible Ag/AgCl electrode before and after sweat test.

As the main anion in the extracellular fluid, chloride ion plays an important role in maintaining acid-base balance, osmotic pressure, water-electrolyte distribution, and muscular activity. In the case of excessive sweating, individuals need to supplement electrolytes in time to avoid dehydration due to a large loss of sodium chloride [53]. Therefore, it is of great significance to detect the content of electrolyte salts in the sweat of individuals with involuntary mass perspiration. In addition, chloride ion in sweat is accepted as a biomarker for diagnosis and prognostic tracking of cystic fibrosis [54]. The above various electrochemical characterizations have confirmed that the ISM-free anion-ISEs could be realized by using insoluble silver salts as both functions of SC and ion recognition. All the above electrodes were fabricated on the solid Ag column electrode. Finally, we examined the feasibility of its flexibility and application in a wearable sensor for sweat analysis. As shown in Figure 4a, the flexible Ag/AgCl electrode was fabricated in three simple steps. The silver was first sputtered on the PET substrate, and then the surface was chemically oxidized into AgCl by FeCl_3_. Finally, the RM solution was drop-casted on the Ag/AgCl as the solid reference electrode (RE), and another bare Ag/AgCl was used for the working electrode (WE). The electrode could undergo 120° bending, which could be satisfied for practical human body test (Figure 4b). On the bending state, the electrode also disclosed Nernstian responses, and the calibration curves were basically coincident (Figure 4c). In addition, since there are some other components in sweat and its pH value is about 4–8, the anti-interference ability of the flexible Ag/AgCl electrode was further carried out. The experimental results in Figure S2 show that the electrode is free from interferences by other components of sweat. As for the pH interference, the total potential for the Ag/AgCl electrode varies basically within 5 mV upon pH value changing from 4 to 8, which is an acceptable fluctuation.

The volunteer ran on a treadmill at a graded load for a period. Figure 4d shows the change of Cl^−^ concentration in human sweat measured by the wearable Ag/AgCl electrode. When the potential was basically stable within a range, the average concentration of Cl^−^ was about 80 mM (Figure 4d), which is consistent within the concentration of sweat Cl^−^ (~10–100 mM). During the stage of cooling down, the concentration of Cl^−^ increases gradually due to the evaporation of sweat (Figure 4d). After cooling down, the sweat sample was collected and analyzed by the ion chromatograph. The result demonstrates that the wearable Ag/AgCl electrode has a high accuracy for the determination of Cl^−^ concentration in human sweat with a relative error of 4% (Figure 4e). The calibration process of the wearable Ag/AgCl electrode was performed immediately after sweat measurement. The corresponding potential response and calibration curves nearly overlapped (Figure 4f and Figure S3). It was demonstrated that the wearable Ag/AgCl electrode was stable even if it was soaked in sweat. This is a crucial indicator, indicating that the data analyzed in real-time by the electrodes can be considered reasonably accurate, regardless of the wearing individual. In the future, the Ag/AgCl electrode can be integrated with other ISM-free SC-ISEs for analyzing other ions, such as Na^+^, K^+^, which can be used for simultaneous and real-time detection of multiple ions in human sweat.

State-of-the-art polymer ISM-based SC-ISEs have been widely used for ion detection in many complex environments. However, the ISM faces a few challenges in many aspects, for example, the biocompatibility of ISM components, water layer effect, and low mechanical strength [5]. Recently, we have proposed an ISM-free concept for SC-ISE based on lithium-ion battery materials [49]. In addition to these materials, biocompatible, redox-active polymers based on carbon nanotubes and covalent functionalized organic matrices exhibit the ion-to-electron transduction ability, and ion recognition could have the potential to construct ISM-free ion sensors [55,56,57].

## 4. Conclusions

Anion sensing has been a long-lasting challenge in solid ion recognition chemistry. This work re-emphasized the classic silver/silver insoluble salts electrodes for constructing ISM-free SC-ISEs. The results were beyond traditional ISM-based SC-ISEs. It exhibited comparable Nernst slope, selectivity, and LOD. The response time, potential stability, and anti-interference ability are superior to the electrode with ISM. Of importance is that this ISM-free electrode has been expanded to other single and multi-valence anions to overcome the lack of anion ionophores in traditional ISM-based SC-ISEs. Finally, the ISM-free electrode exhibited good flexibility and was successfully applied to the on-body analysis of Cl^−^ concentration in human sweat. In the future, these ISM-free anion electrodes can be explored in more complex environments, such as marine ion sensing.

## Data Availability

The data are available upon reasonable request from the corresponding author.

## Supplementary Materials

The following are available online at https://www.mdpi.com/article/10.3390/membranes11120959/s1. Figure S1: Selectivity coefficient testing of the prepared Cl^–^−ISEs: potentiometric responses of the Ag/AgCl electrode (blue line) and the ISM electrode (orange line) in aqueous solution from 0.1 μM to 0.1 M: (a) KHCO_3_, (b) KH_2_PO_4_, (c) K_2_HPO_4_, (d) K_2_CO_3_, (e) K_2_SO_4_, and (f) K_3_PO_4_. Figure S2: pH (a) and metabolite (b) interference tests of the prepared flexible Ag/AgCl electrode. Figure S3: Potential response curves of the flexible Ag/AgCl electrode before (black line) and after (red line) sweat test. Table S1: Comparison of Ag/AgCl-based Cl^−^ sensors with previously reported sensors, Table S2: A comparison of Cl^−^ sensors between Ag/AgCl and ISM-based SC-ISEs of Cl^–^. References [30,38,45,46,58,59,60,61,62,63,64,65,66,67] have been cited in Supplementary Materials.
