# Supplementary material for: Solid-Contact Potentiometric Anion Sensing Based on Classic Silver/Silver Insoluble Salts Electrodes without Ion-Selective Membrane
Source: Membranes (Basel). 2021 Dec 5;11(12):959. doi: 10.3390/membranes11120959 (PMC8707216; doi:10.3390/membranes11120959)
Supplement: Supplementary file 1 [file membranes-11-00959-s001.zip › membranes-1471659-supplementary.pdf]

*membranes*

MDPI

*Supplementary Materials*

# Solid-Contact Potentiometric Anion Sensing Based on Classic Silver/Silver Insoluble Salts Electrodes without Ion-Selective Membrane

**Chunxian Liao [1], Lijie Zhong [1], Yitian Tang [1,2], Zhonghui Sun [1], Kanglong Lin [1], Longbin Xu [1,2], Yan Lyu [1,2], Dequan He [1], Ying He [1], Yingming Ma [1], Yu Bao [1], Shiyu Gan [1,*] and Li Niu [1]**

[1] Guangzhou Key Laboratory of Sensing Materials & Devices, Center for Advanced Analytical Science, School of Chemistry and Chemical Engineering, Guangzhou University, Guangzhou 510006, China; gdcxliao@e.gzhu.edu.cn (C.L.); ccljzhong@gzhu.edu.cn (L.Z.); yitiantang@e.gzhu.edu.cn (Y.T.); cczhsun@gzhu.edu.cn (Z.S.); longkanglin@e.gzhu.edu.cn (K.L.); longbinx@gzhu.edu.cn (L.X.); yanlyu@e.gzhu.edu.cn (Y.L.); dequanhe@e.gzhu.edu.cn (D.H.); ccyhe@gzhu.edu.cn (Y.H.); ccymma@gzhu.edu.cn (Y.M.); baoyu@gzhu.edu.cn (Y.B.); lniu@gzhu.edu.cn (L.N.)

[2] School of Civil Engineering, Guangzhou University, Guangzhou 510006, China

* Correspondence: ccsygan@gzhu.edu.cn

**Citation:** Liao, C.; Zhong, L.; Tang, Y.; Sun, Z.; Lin, K.; Xu, L.; Lyu, Y.; He, D.; He, Y.; Ma, Y.; et al. Solid-Contact Potentiometric Anion Sensing Based on Classic Silver/Silver Insoluble Salts Electrodes without Ion-Selective Membrane. *Membranes* **2021**, *11*, 959. https://doi.org/10.3390/membranes11120959

Academic Editor: Beata Paczosa-Bator

Received: 4 November 2021
Accepted: 28 November 2021
Published: 5 December 2021

**Publisher's Note:** MDPI stays neutral with regard to jurisdictional claims in published maps and institutional affiliations.

**Copyright:** © 2021 by the authors. Licensee MDPI, Basel, Switzerland. This article is an open access article distributed under the terms and conditions of the Creative Commons Attribution (CC BY) license (https://creativecommons.org/licenses/by/4.0/).

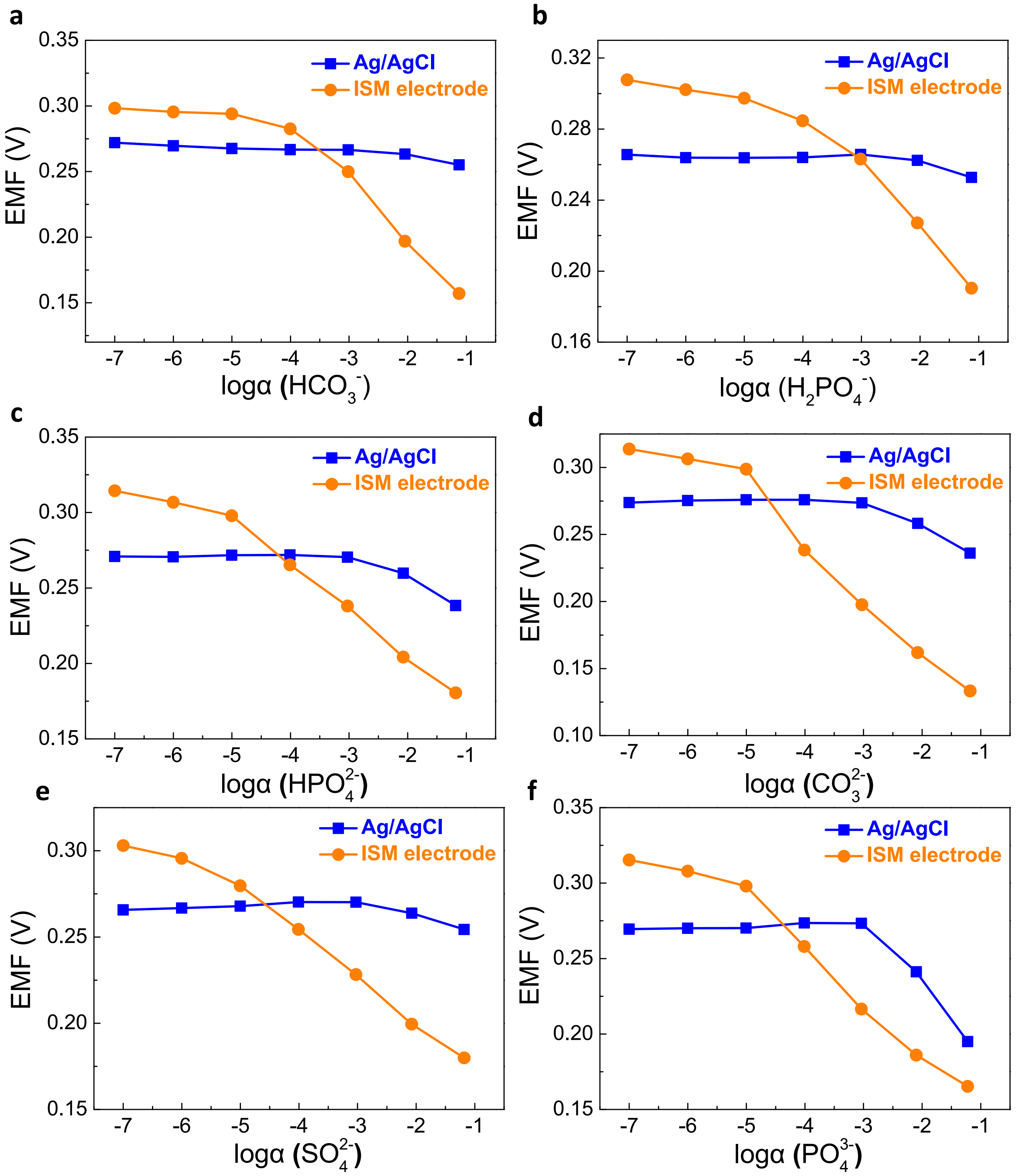


**Figure S1.** Selectivity coefficient testing of the prepared $Cl^-$–ISEs: potentiometric responses of the Ag/AgCl electrode (blue line) and the ISM electrode (orange line) in aqueous solution from 0.1 μM to 0.1 M: (**a**) $KHCO_3$, (**b**) $KH_2PO_4$, (**c**) $K_2HPO_4$, (**d**) $K_2CO_3$, (**e**) $K_2SO_4$, and (**f**) $K_3PO_4$.

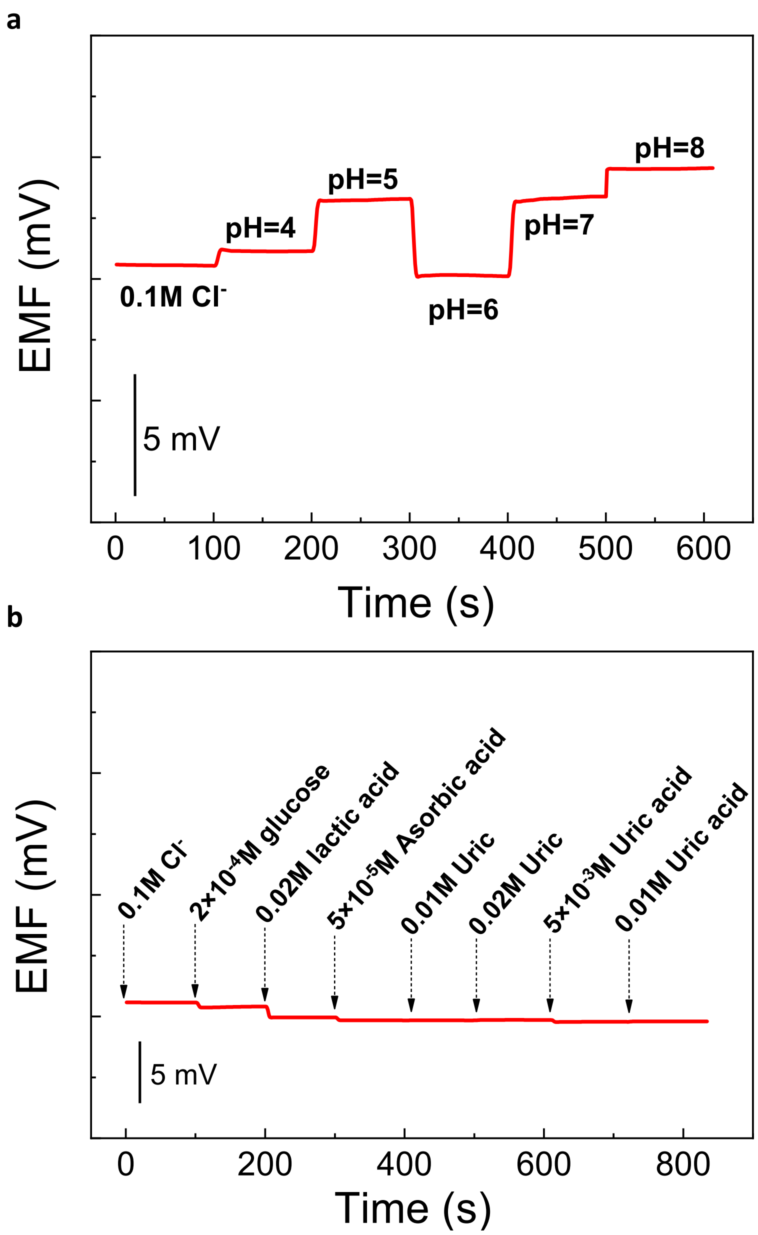


**Figure S2.** pH (**a**) and metabolite (**b**) interference tests of the prepared flexible Ag/AgCl electrode.

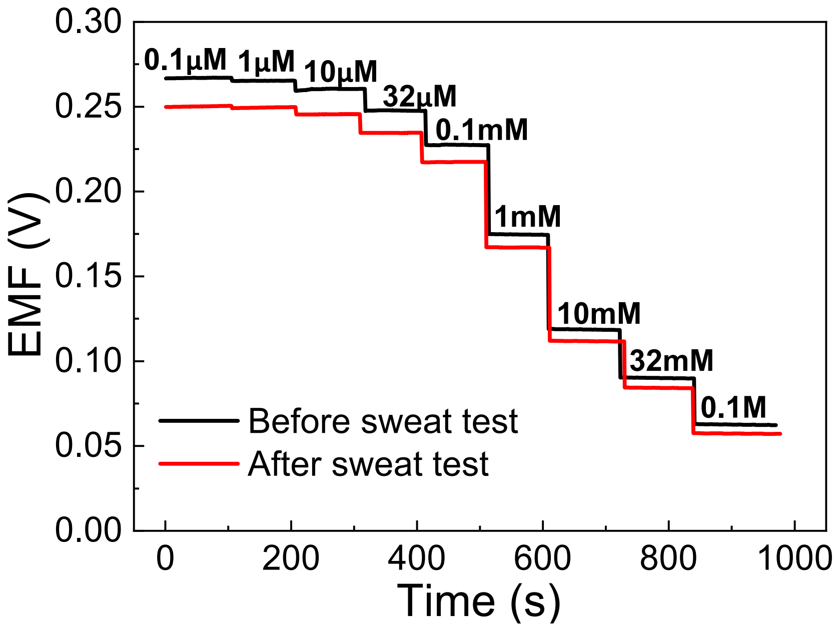


**Figure S3.** Potential response curves of the flexible Ag/AgCl electrode before (black line) and after (red line) sweat test.

**Table S1.** Comparison of Ag/AgCl-based $Cl^-$ sensors with previously reported sensors.

| Preparation methods | $Cl^-$ range (Log $\alpha$) | LOD (Log $\alpha$) | Sensitivity (mV/dec) | Response time | Stability (mV/h) | Ref. |
|---|---|---|---|---|---|---|
| Anodizing | −2.7–0.48 | - | −59.0 ± 1.0 | - | - | [1] |
| Coulometric chloridization | −4.3–−1 | - | −53.0 ± 1.5 | - | - | [2] |
| Ag/AgCl paste | −3–0.48 | - | −58 | - | - | [3,4] |
| Anodizing | −2.7–0.6 | - | −59.0 ± 2.0 | - | - | [5] |
| $FeCl_3$ oxidation | −3–−1 | - | −63.0 ± 4.0 | - | - | [6] |
| $FeCl_3$ oxidation | −2–−1 | - | −52.8 ± 0.7 | - | - | [7] |
| Ag/AgCl conductive ink | −2–0 | −3.3 | −51.5 ± 2.9 | 30s | 0.24 | [8] |
| Anodizing | −3–0 | -- | −64 | - | - | [9] |
| Constant current oxidation | −4.5–−1 | −4.8 | −57.1 ± 1.2 | 25s | 0.033 | This work |

**Table S2.** A comparison of $Cl^-$ sensors between Ag/AgCl and ISM-based SC-ISEs of $Cl^-$.

| SC-ISEs of $Cl^-$ | $Cl^-$ range (Log $\alpha$) | LOD (Log $\alpha$) | Sensitivity (mV/dec) | Response time | Stability (mV/h) | Ref |
|---|---|---|---|---|---|---|
| PPy/ISM | −4–−1 | −4.7 | −48.4 ± 0.73 | - | - | [10] |
| PANI/ISM | −4–−1 | −4.6 | −58.8 ± 1.3 | - | - | [11] |
| PEDOT/ISM | −3–−1 | −3.9 | −62.7 ± 0.1 | - | - | [12] |
| POT/ISM | −3–−1 | - | −53.35 ± 0.29 | - | - | [13] |
| $RuO_2$-GO/ISM | −5–−1 | - | −44.45 | - | - | [14] |
| Ag/AgCl | −4.5–−1 | −4.8 | −57.1 ± 1.2 | 25 s | 0.033 | This work |

## References

1. Angst, U.; Elsener, B.; Larsen, C.K.; Vennesland, Ø. Potentiometric determination of the chloride ion activity in cement based materials. *J. Appl. Electrochem.* **2009**, *40*, 561–573, https://doi.org/10.1007/s10800-009-0029-6.
2. Cranny, A.; Harris, N.; White, N. Screen Printed Potentiometric Chloride Sensors. *Procedia Eng.* **2014**, *87*, 220–223, https://doi.org/10.1016/j.proeng.2014.11.626.
3. Dam, V.A.T.; Zevenbergen, M.A.G.; Schaijk, R. van. Flexible Chloride Sensor for Sweat Analysis. *Procedia Eng.* **2015**, *120*, 237–240, https://doi.org/10.1016/j.proeng.2015.08.588.

4. Dam, V.A.T.; Zevenbergen, M.A.G.; Schaijk, R. van. Toward wearable patch for sweat analysis. *Sens. Actuators B: Chem.* **2016**, *236*, 834–838, https://doi.org/10.1016/j.snb.2016.01.143.
5. Femenias, Y. S.; Angst, U.; Caruso, F.; Elsener, B. Ag/AgCl ion-selective electrodes in neutral and alkaline environments containing interfering ions. *Mater. Struct.* **2016**, *49*, 2637–2651, https://doi.org/10.1617/s11527-015-0673-8.
6. Abbas, Y.; Zhu, X.; de Boer, H. L.; Tanvir, N. B.; Olthuis, W.; van der Berg, A. Potentiometric measurement with a Kelvin probe: Con-tactless measurement of chloride ions in aqueous electrolyte. *Sens. Actuators B: Chem.* **2016**, *236*, 1126–1132, https://doi.org/10.1016/j.snb.2016.06.150.
7. Choi, D.H.; Li, Y.; Cutting, G.R.; Searson, P.C. A wearable potentiometric sensor with integrated salt bridge for sweat chloride measurement. *Sens. Actuators B: Chem.* **2017**, *250*, 673–678, https://doi.org/10.1016/j.snb.2017.04.129.
8. Xu, G.; Cheng, C.; Yuan, W.; Liu, Z.; Zhu, L.; Li, X.; Lu, Y.; Chen, Z.; Liu, J.; Cui, Z.; et al. Smartphone-based battery-free and flexible electrochemical patch for calcium and chloride ions detections in biofluids. *Sens. Actuators B: Chem.* **2019**, *297*, 126743, https://doi.org/10.1016/j.snb.2019.126743.
9. Tian, Y.; Zhang, P.; Zhao, K.; Du, Z.; Zhao, T. Application of Ag/AgCl Sensor for Chloride Monitoring of Mortar under Dry-Wet Cycles. *Sensors* **2020**, *20*, 1394, https://doi.org/10.3390/s20051394.
10. Zielińska, R.; Mulik, E.; Michalska, A.; Achmatowicz, S.; Maj-Żurawska, M. All-solid-state planar miniature ion-selective chloride electrode. *Anal. Chim. Acta* **2002**, *451*, 243–249, https://doi.org/10.1016/S0003-2670(01)01407-6.
11. Paciorek, R.; Bieganowski, P.; Maj-Żurawska, M. Miniature planar chloride electrodes. *Sens. Actuators B: Chem.* **2005**, *108*, 840–844, https://doi.org/10.1016/j.snb.2004.11.071.
12. Sjöberg-Eerola, P.; Bobacka, J.; Lewenstam, A.; Ivaska, A. All-solid-state chloride sensors based on electronically conducting, semiconducting and insulating polymer membranes. *Sens. Actuators B: Chem* **2007**, *127*, 545–553, https://doi.org/10.1016/j.snb.2007.05.004.
13. Sjöberg-Eerola, P.; Nylund, J.; Bobacka, J.; Lewenstam, A.; Ivaska, A. Soluble semiconducting poly(3-octylthiophene) as a solid-contact material in all-solid-state chloride sensors. *Sens. Actuators B: Chem.* **2008**, *134*, 878–886, https://doi.org/10.1016/j.snb.2008.06.041.
14. Tseng, S.; Wu, T.; Chou, J.; Liao, Y.; Lai, C.; Chen, J.; Yan, S.; Huang, M.; Tseng, T.; Nien, Y. Research of sensing characteristic and dynamic measurement of graphene oxides modified flexible arrayed $RuO_2$ chlorine ion sensor. *Mater. Res. Bull.* **2018**, *101*, 155–161, https://doi.org/10.1016/j.materresbull.2018.01.015.
